# Wnt5a/CaMKII/ERK/CCL2 axis is required for tumor-associated macrophages to promote colorectal cancer progression

**DOI:** 10.7150/ijbs.40535

**Published:** 2020-02-04

**Authors:** Qing Liu, Jialin Song, Yue Pan, Dongdong Shi, Chaogang Yang, Shuyi Wang, Bin Xiong

**Affiliations:** 1 Department of Gastrointestinal Surgery & Department of Gastric and Colorectal Surgical Oncology, Zhongnan Hospital of Wuhan University, Wuhan 430071, China.; 2 Hubei Key Laboratory of Tumor Biological Behaviors, Wuhan 430071, China.; 3 Hubei Cancer Clinical Study Center, Wuhan 430071, China.; 4 Department of Intensive Care Unit, Zhongnan Hospital of Wuhan University, Wuhan 430071, China.

**Keywords:** tumor-associated macrophages, colorectal cancer, Wnt5a, CCL2

## Abstract

Tumor-associated macrophages (TAMs) are closely correlated with tumor occurrence, invasion, and metastasis. However, factors affecting the biological functions of TAMs in colorectal cancer (CRC) are incompletely understood. Here, we found that Wnt5a was mainly expressed on TAMs of tumor stroma but not on CRC cells. Subsequently, we found that Wnt5a^+^ TAMs facilitated tumor cell proliferation and migration, and recruited macrophages infiltration. Furthermore, Wnt5a knockdown impaired the pro-tumor roles of TAMs *in vivo* and *in vitro*. Mechanistically, the cancer-promoting roles of Wnt5a in TAMs depended on CaMKII-ERK pathway-mediated CCL2 secretion. Our data reveal the crucial role played by TAM-expressed Wnt5a in CRC tumorigenesis through paracrine secretion of CCL2. We first report the connection between Wnt5a/CaMKII/ERK/CCL2 axis and biological functions of TAMs in tumor microenvironment, indicating that Wnt5a may be a novel therapeutic target for CRC.

## Introduction

Tumor microenvironment (TME), mainly composed of tumor cells, immune cells, cancer-associated fibroblasts and the extracellular matrix, is closely related to the occurrence, growth and metastasis of cancers 1. The most abundant immune cells in TME are macrophages, referred to as tumor-associated macrophages (TAMs), of which the functions are determined by their polarization state in TME 2, 3. In general, TAMs are mainly categorized into classically activated macrophages (M1 phenotype) and alternatively activated macrophages (M2 phenotype) 1, 4. M1-like TAMs, highly expressing HLA-DR, CD86, IL-1β, IL-12, IL-23 and INOS, are thought to induce inflammatory response and activate anti-tumor immune response, resulting in tumor suppression 5, 6. Whereas M2-like TAMs, of which representative markers are CD206, CD163, IL-10, TGFβ, CCL17, CCL18, CCL22 and Arg-1, exert anti-inflammatory activities and play a crucial role in promoting tumor invasion and metastasis 3, 7. As TAMs play a central role in cancer progression, our team studied the crosstalk between tumor cells and TAMs. We found that TAMs, especially M2-like TAMs, induced epithelial-mesenchymal transition of tumor cells to promote CRC growth, invasion and metastasis 8. Although the pro-tumor roles of TAMs are well studied, the factors affecting the biological function of TAMs in CRC are incompletely understood.

Wnt family, including 19 members, is involved in the regulation of tumor initiation and development processes through activation of canonical Wnt/β-catenin pathway and non-canonical pathways 9, 10. Wnt5a, a member of Wnt family, plays various physiological roles in tumor growth, invasion and metastasis, mainly by activating the non-canonical pathways such as planar cell polarity (PCP), JNK and Ca2^+^/CaKMII pathways 11, 12. Although the roles of Wnt5a on tumor cells have been well studied in multiple solid tumors 11, 13, 14, little is known about the interaction between Wnt5a and TME in colorectal cancer (CRC). Lee et al. found that TAMs were crucial for Wnt5a-mediated castration-resistant prostate cancer 15. It indicated that Wnt5a might facilitate tumor progression by affecting the biological function of TAMs. In fact, Smith and colleagues reported a long time ago that Wnt5a^+^ cells in CRC were proven to be TAMs 16. However, how Wnt5a regulates the biological function of TAMs in CRC remains largely unknown.

Here, we found that Wnt5a was mainly expressed in TAMs of tumor stroma, especially M2-like TAMs. Moreover, our study showed that Wnt5a^+^ TAMs promoted tumor cell proliferation and migration, and recruited macrophages infiltration. Knockdown of Wnt5a in TAMs reduced the tumorigenic capacities of CRC cells *in vitro* and *in vivo*. Mechanistically, pro-tumor functions of Wnt5a in TAMs depended on CaMKII-ERK pathway-mediated CCL2 secretion. Taken together, these data indicate that Wnt5a might be a new functional TAM marker, providing a novel insight into targeting Wnt5a in cancer therapy.

## Materials and methods

### Cells and tissues

Human THP-1 monocytes, Human CRC cell lines (HCT116, DLD-1) were purchased from ATCC. Cells were maintained in RPMI 1640 medium (Hyclone, USA) containing 10% heat-inactivated fetal bovine serum (Gibco, USA) and cultured at 37°C in a humidified air with 5% CO2. A total of 10 CRC specimens were obtained from patients who underwent curative resection of primary CRC at Zhongnan Hospital, Wuhan University (Wuhan, China). Informed consents were collected from these enrolled patients. Human recombinant Wnt5a and CCL2 protein were obtained from R&D Systems, and the final concentration of 100ng/ml and 50ng/ml were used respectively. U0126 (p-ERK inhibitor, Merck), Box5 (Wnt5a inhibitor, Merck), CK59 (p-CaMKII inhibitor, Merck) were used at 20ng/ml, 100μM, 50μM, respectively. Human neutralizing CCL2 antibody was obtained from Biolegend, USA.

### Immunohistochemistry and immunofluorescence staining

After dewaxing and antigen retrieval, the paraffin-embedded sections were blocked in 3% BSA and then incubated with monoclonal antibodies against human Wnt5a (1:100, Abcam), CD68 (1:100, Abcam), CCL2 (1:100, CST), Ki67 (1:500, Abcam) at 4°C overnight. Immunoreactions were visualized with DAB. For Immunofluorescence staining, the used primary antibodies are as follows: human Wnt5a (1:100, Abcam, USA), CD68 (1:100, Abcam, USA), CD163 (1: 100, Abcam, USA), HLA-DR (1:100, CST, USA). Subsequently, sections were stained with fluorochrome-conjugated secondary antibodies and nucleus staining was conducted using DAPI.

### THP-1 cell differentiation and macrophage generation

THP-1 monocyte was treated with 100 ng/ml PMA (Sigma, USA) for 24h to differentiate into THP-1 macrophage (M0 macrophage), which then incubated with LPS (100 ng/ml)/IFN-γ (20 ng/ml) or IL-4 (50 ng/ml)/IL-13 (20 ng/ml) for 48h to generate M1 or M2 macrophage. For mimicking TAMs, THP-1 macrophages (M0 macrophages) were co-cultured with HCT116 or DLD-1 cells in a transwell co-cultivation system with 0.4μm pore size (Corning, USA). After 48h, the co-cultured M0 macrophages were harvested to obtain TAMs.

### Real-time quantitative PCR (RT-qPCR)

Total RNA was extracted using the Trizol reagent (Invitrogen, USA) according to the manufacturer's instructions. 3ug RNA was reverse transcribed to cDNA by Primescript™ RT reagent Kit (Vazyme, China). RT-qPCR was performed using the SYBR-Green PCR Master Mix (Vazyme, Nanjing, China). Relative mRNA expression level was calculated using 2^-ΔΔCt^ method. The primer sequences are listed in Table S1.

### Western blot

Western blot was performed according to the previous report 8. Briefly, total proteins were separated by 10% SDS-PAGE gels and transferred to a PVDF membrane. After blocking with 5% skim milk, the PVDF membrane was incubated with primary antibodies and HRP-conjugated secondary antibodies. The primary antibodies are as follows: Wnt5a (1:1000, Abcam), p-ERK1/2 (1:1000, CST), ERK1/2 (1:1000, Protein Tech), p-CaMKII (1:1000, CST), CaMKII (1:1000, Protein Tech), α-Tubulin (1:5000, Protein Tech), GAPDH (1:5000, Protein Tech).

### Cell transfection

Plasmid vectors of Wnt5a-shRNA, negative control RNA (sh-NC) were chemically synthesized, constructed, and embedded with Lentivirus by GenePharma (Shanghai, China). Then Wnt5a expression in TAMs was knocked down by transduction with sh-Wnt5a or sh-NC.

### Conditioned medium collection

5×10^5^ TAMs were transfected with sh-Wnt5a or sh-NC for 8h and then washed 3 times with PBS, and subsequently cultured with 1ml fresh serum-free medium. After 24 hours, the cultured supernatant was harvested and centrifuged for 10 minutes at 10,000 r/min, which was defined as conditioned medium (CM). Then CM mixed with 10% serum culture medium at a 1:1 ratio was used to treat CRC cells.

### CCK-8 assay

Cell proliferation was assessed with a CCK-8 reagent kit (Vazyme, China). 5000 HCT116 or DLD-1 cells were seeded in 96-well plates and incubated with the conditioned media for the indicated time. Then CCK-8 solution was added into each well and the spectral absorbance was measured. Each experiment was carried out in triplicates.

### Migration and chemotaxis assays

These experiments were conducted using a 24-well transwell system with 8μm pore size (Corning, USA). For the migration assay, 5×10^4^ CRC cells were seeded into the upper chamber and cultured in serum-free medium. The conditioned medium was added into the lower chamber. After co-culture for 24h, the chamber was fixed in 4% paraformaldehyde and then stained using 0.1% crystal violet (Sigma). All cells in the upper chamber were removed using a cotton swab. Cells in the lower chamber were photographed and counted at 5 randomly microscopic fields. For the chemotaxis assay, 5×10^4^ THP-1 macrophages were seeded into the upper chamber, and the remaining steps were similar to the migration assay. Each experiment was carried out in triplicates.

### ELISA

The secretion level of CCL2 was measured by an ELISA kit (R&D Systems, USA) according to the manufacturer's instructions. Every experiment was performed in triplicates.

### *In vivo* experiments

A total of 15 BALB/c nude mice (female, 4-5 weeks old) were obtained from the Hubei Research Center of Laboratory Animals (Wuhan, China). All xenograft experiments were performed strictly according to the guidelines of the Institutional Animal Care and Use Committee at Wuhan University. Every mouse was injected intravenously with the liposomal clodronate for macrophage depletion as reported previously by our group 17. BALB/c nude mice were randomly distributed into 3 groups (n=5 per group). HCT116 cells (1×10^6^), HCT116 cells (1×10^6^)+sh-NC TAMs (5×10^5^), HCT116 cells (1×10^6^)+sh-Wnt5a TAMs (5×10^5^) were separately resuspended with 150ul PBS, and then subcutaneously inoculated into the right flank of nude mice. After cell inoculation, THP-1 macrophages were injected into the caudal veins at the dose of 10^6^ cells/50μL per mouse every 4 days for eight times 17. After 7 days, the tumor size was measured using the digital Vernier Caliper every 5 days. The tumor volume was computed using the formula: volume = length × width^2^/2. After 32 days, BALB/c mice were sacrificed. Tumor tissues were harvested and calculated for weight and volume, and then further analysis by IHC staining or RT-qPCR.

### Statistics analysis

Statistical analyses were performed with SPSS (version 19.0, IBM, USA). All presented results were shown as means ± SEM from at least 3 independent experiments. Means of continuous variables were appropriately tested with two-tailed Student's t-test or one-way analyses of variance. p values <0.05 were considered statistically significant.

## Results

### Wnt5a was mainly localized in TAMs, especially M2-like TAMs

**Figure 1:**
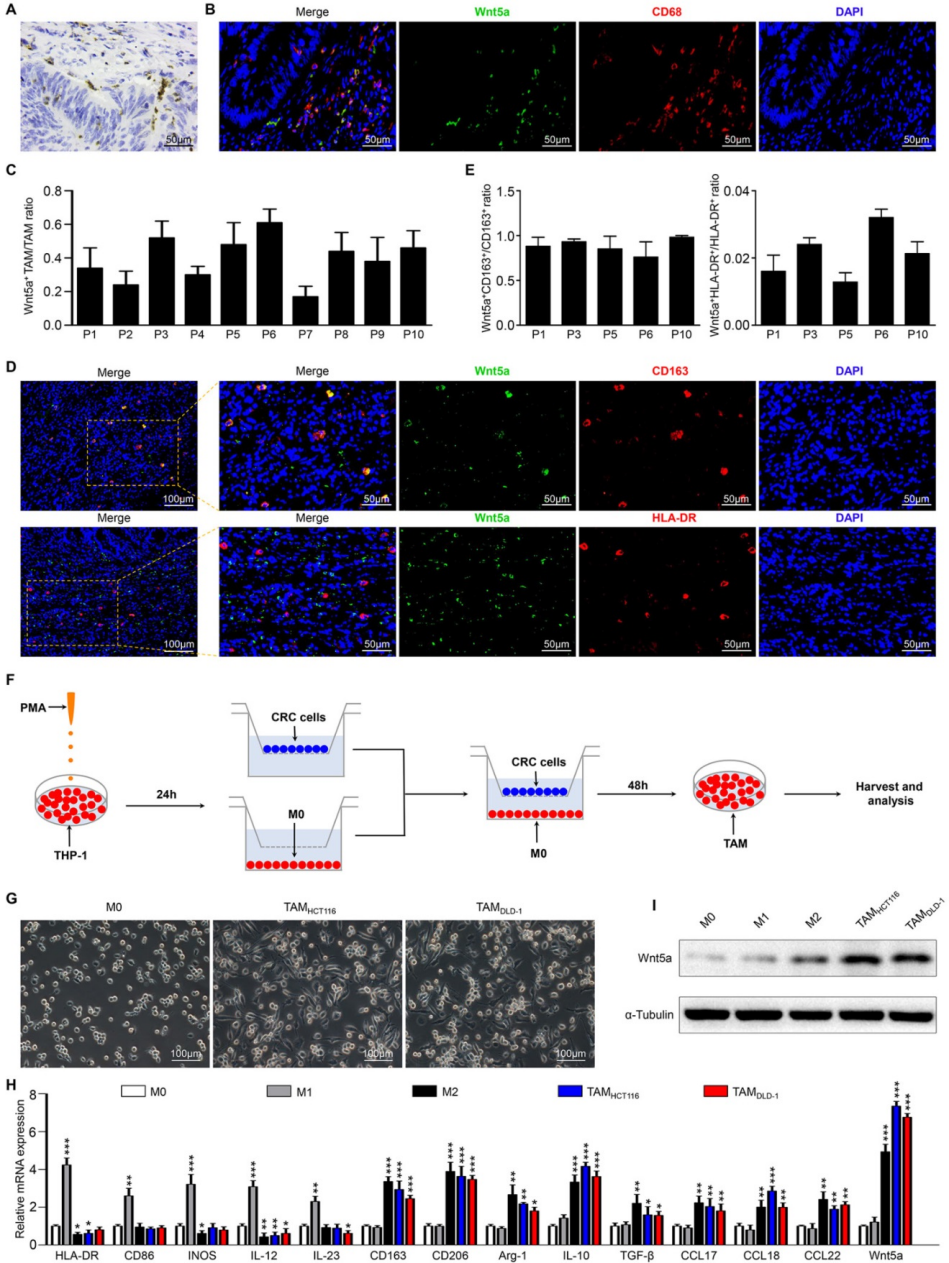
** Wnt5a is mainly localized in TAMs of tumor stroma. A** Representative IHC staining photographs for Wnt5a in CRC tissues. Bar = 50μm. **B** Representative immunofluorescence photographs for Wnt5a, CD68, DAPI in CRC samples. Bar = 50μm. **C** Quantitative analysis of Wnt5a^+^ TAM/TAM ratio in 10 CRC samples. The number of Wnt5a^+^ TAM and TAM was counted manually in at least 10 fields (400× magnification) for each section. **D** Representative immunofluorescence staining images for Wnt5a, CD163, HLA-DR, DAPI in CRC specimens. Bar = 50μm. **E** Quantitative analysis of Wnt5a^+^CD163^+^/CD163^+^ TAM ratio and Wnt5a^+^HLA-DR^+^/HLA-DR^+^ TAM ratio in 5 CRC samples. The number of Wnt5a^+^CD163^+^ TAM, CD163^+^ TAM, Wnt5a^+^HLA-DR^+^ TAM and HLA-DR^+^ TAM was counted manually in at least 10 fields (400× magnification) for each section. **F** Flow chart of mimicking TAMs. **G** Representative bright-field images of M0 macrophages and TAMs. Bar = 100μm. **H** Relative mRNA expression of M1 markers (HLA-DR, CD86, INOS, IL-12, IL-23), M2 markers (CD163, CD206, Arg-1, IL-10, TGFβ, CCL17, CCL18, CCL22), and Wnt5a in M0 macrophages, M1 macrophages, M2 macrophages and TAMs. Error bars, SEM. **I** Wnt5a expression in M0 macrophages, M1 macrophages, M2 macrophages and TAMs. *P<0.05. **P< 0.01. ***P<0.001

We observed that Wnt5a was primarily localized in the tumor stroma but not on tumor cells (Fig. 1A). we focused on TAMs, a vital type of the most dynamic immune cells in the tumor stroma. To further investigate the correlation between Wnt5a expression and TAMs, sections of human colorectal cancer tissues were stained to examine the expression of CD68 (a pan-macrophage marker) and Wnt5a. Interestingly, Wnt5a was primarily co-expressed with CD68 in CRC tissues (Fig. 1B). We found that about 17%-61% TAMs were Wnt5a^+^ cells in different CRC specimens (Fig.1C). It indicated that not all TAMs expressed Wnt5a. It is well-known that TAM is mainly categorized as M1-like or M2-like phenotype, so we suspected that Wnt5a^+^ TAMs might be associated with M1-like or M2-like TAM subtype. Then 5 CRC samples with relatively high Wnt5a^+^ TAM/TAM ratio were further subject to detection of M1 (HLA-DR) and M2 (CD163) makers. Intriguingly, we found that Wnt5a was co-localized with CD163, while not HLA-DR (Fig. 1D), which was also confirmed by quantitative analysis (Fig. 1E). These data indicate that Wnt5a^+^ TAM is a subtype of M2-like TAM.

To confirm the above results, an *in vitro* model of tumor-associated macrophages (TAMs) was applied as reported previously 18. As shown in the schematic (Fig. 1F), human monocyte cell line THP-1 was treated with PMA to generate THP-1 macrophages (M0 macrophages), which were then co-cultured with HCT116 or DLD-1 cells for 48h to obtain TAMs. Compared with M0 macrophages, the cellular morphology of co-cultured TAMs became stretched and elongated (Fig. 1G), which was similar to M2-like TAMs in the tumor microenvironment 19. The co-cultured TAMs also exhibited higher M2 markers (CD163, CD206, Arg-1, IL-10, TGF-β, CCL17, CCL18, CCL22), and lower M1 markers (HLA-DR, CD86, INOS, IL12, IL-23), which was consistent with IL-4/IL-13-induced M2 macrophages (Fig. 1H). Therefore, TAM produced by the *in vitro* model was a kind of macrophage based on the M2 phenotype. Then we examined Wnt5a expression in different phenotypes of macrophages. As shown in Fig. 1I and H, TAMs and M2 macrophages apparently overexpressed Wnt5a, while M0 and M1 macrophages scarcely expressed Wnt5a. Together, our findings reveal that Wnt5a is primarily expressed in M2-like TAMs.

### Wnt5a^+^ TAMs promote CRC cells proliferation, migration and macrophage recruitment *in vitro*

**Figure 2:**
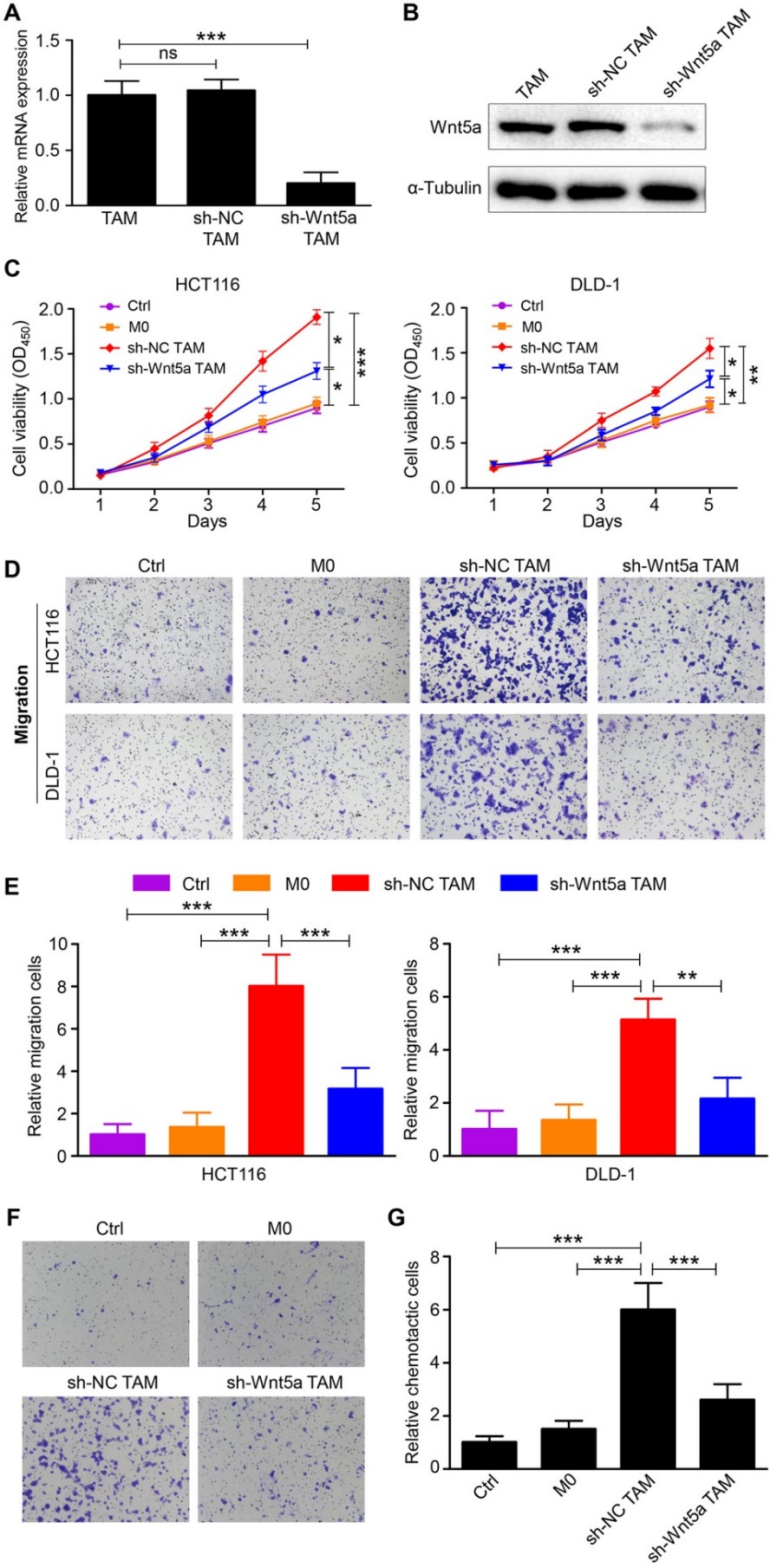
** Wn5a is essential for TAMs to promote CRC cells proliferation, migration and macrophage recruitment. A** RT-qPCR analysis of Wnt5a mRNA expression in Wnt5a^+^ TAM transfected with sh-NC or sh-Wnt5a. Error bars, SEM. **B** Western blot analysis of Wnt5a protein expression in Wnt5a^+^ TAM transfected with sh-NC or sh-Wnt5a. **C** Cell viability of CRC cells cultured with fresh medium (control), supernatant from M0 macrophages, sh-NC or sh-Wnt5a TAMs. Error bars, SEM. **D** Transwell migration assay of CRC cells treated with conditioned medium. (magnification, ×100). **E** Quantification analysis of migratory cells in five fields was counted manually. Error bars, SEM. **F** Chemotaxis analysis of THP-1 macrophages toward fresh medium (control), supernatant from M0 macrophages, sh-NC or sh-Wnt5a TAMs. (magnification, ×100). **G** Quantification analysis of chemotactic cells in five fields was counted manually. Error bars, SEM. *P<0.05. **P< 0.01. ***P<0.001

To assess the role of TAM-expressed Wnt5a in CRC cells, TAMs were transfected with sh-NC or sh-Wnt5a. The transfection efficiency of sh-Wnt5a or sh-NC was estimated by RT-qPCR and western blot (Fig. 2A and B). The effects of fresh medium (control), conditioned medium from M0 macrophages, sh-Wnt5a or sh-NC TAMs on cell proliferation of HCT116 or DLD-1 cells were examined using CCK-8 assay. Results showed that Wnt5a^+^ TAMs enhanced the proliferation capacity of HCT116 cells compared with M0 macrophages, so did DLD-1 cells (Fig. 2C). This enhancement was attenuated by Wnt5a knockdown (Fig. 2C). Moreover, the migratory ability of HCT116 or DLD-1 cells was remarkably improved by Wnt5a^+^ TAMs (Fig. 2D and E). After silencing Wnt5a expression in TAMs, this result was reversed (Fig. 2D and E). These data suggest that Wnt5a is crucial for TAMs to facilitate tumor cells proliferation and migration. Intriguingly, we also observed that THP-1 macrophages were recruited by TAM-expressed Wnt5a (Fig. 2F and G), which could lead to more TAMs infiltration in the tumor microenvironment, thereby resulting in tumor support. Similarly, this recruitment was reduced by knockdown of Wnt5a (Fig. 2F and G).

### Wnt5a doesn't directly influence CRC cells and macrophage recruitment

Wnt5a is a secreted protein that exerts its cellular effects via autocrine or paracrine routes 11. As shown in Figure S1A, the secretion level of Wnt5a in TAMs was around 2.31 ng/ml, which was significantly higher than that of M0 macrophages. Combined with the above results, we hypothesized that the pro-tumor functions of TAMs were mediated through the secretion of Wnt5a. We then treated CRC cells directly with human recombinant protein Wnt5a and observed the direct effect of Wnt5a on tumor cells. Unexpectedly, we found that Wnt5a hardly affected the cell viability of HCT116 or DLD-1 cells (Figure S1B). In the transwell migration assay, Wnt5a did not promote the migration ability of HCT116 or DLD-1 cells (Figure S1C and D). Consistently, Wnt5a also failed to recruit macrophages (Figure S1E and F). Together, these data demonstrate that Wnt5a does not directly promote tumor progression, and is not a macrophage chemokine.

### Wnt5a^+^ TAMs exert tumor-promoting effects via CCL2

**Figure 3:**
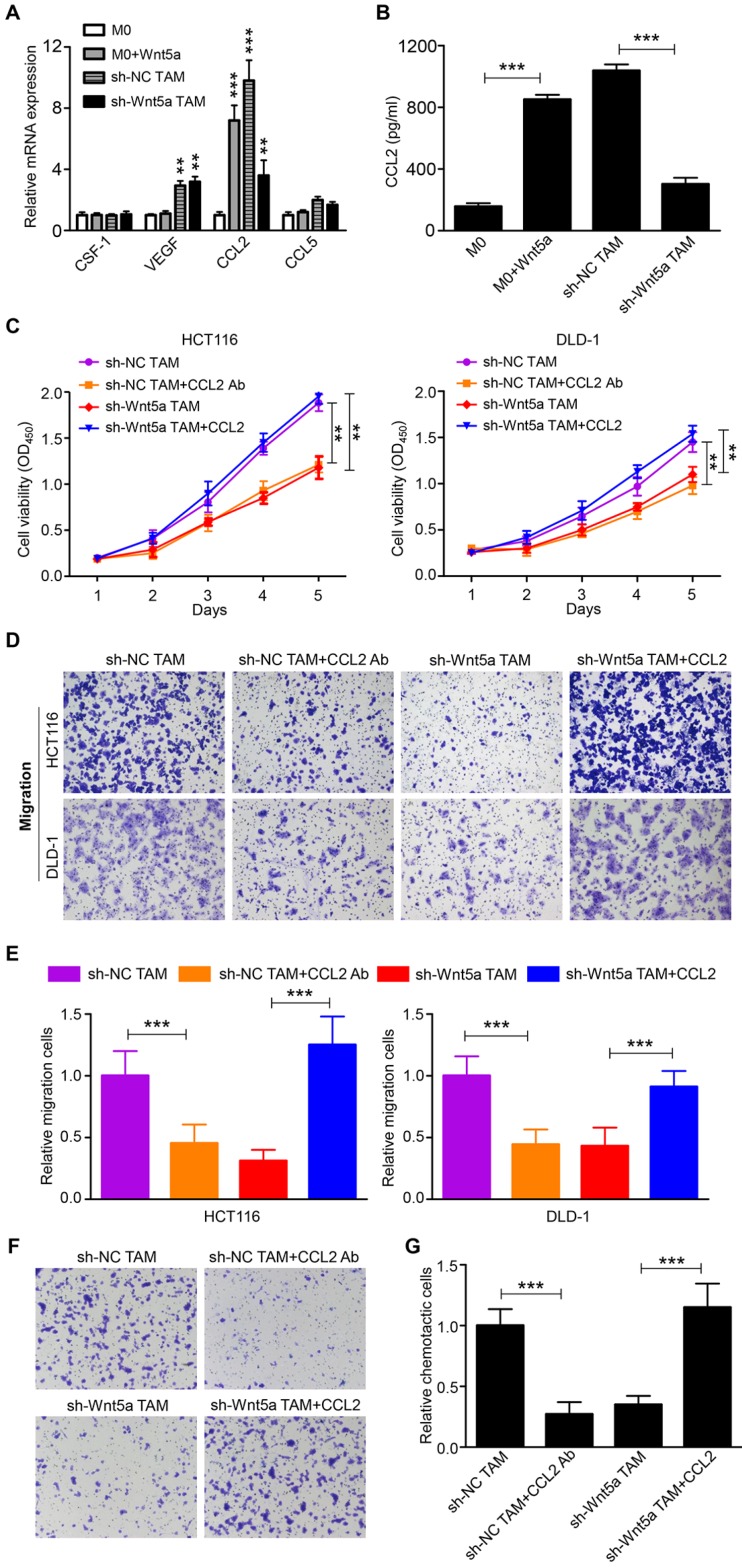
** Wnt5a^+^ TAMs exert tumor-promoting effects via CCL2. A** The potential cytokines in Wnt5a^+^ TAMs or Wnt5a-treated M0 macrophages, which promoted tumor progression and macrophage infiltration, were identified by RT-qPCR. Error bars, SEM. **B** CCL2 secretion level in Wnt5a^+^ TAMs or Wnt5a-treated M0 macrophages was detected by ELISA. Error bars, SEM. **C** Cell viability of CRC cells treated with sh-NC TAM supernatant with or without CCL2 Ab, sh-Wnt5a TAM supernatant with or without recombinant CCL2. Error bars, SEM. **D** Transwell migration assay of CRC cells treated with sh-NC TAM supernatant with or without CCL2 Ab, sh-Wnt5a TAM supernatant with or without recombinant CCL2. (magnification, ×100). **E** Quantification analysis of migratory cells in five fields was counted manually. Error bars, SEM. **F** Chemotaxis analysis of THP-1 macrophages toward sh-NC TAM supernatant with or without CCL2 Ab, sh-Wnt5a TAM supernatant with or without recombinant CCL2. (magnification, ×100). **G** Quantification analysis of chemotactic cells in five fields was counted manually. Error bars, SEM. **P< 0.01. ***P<0.001

Although Wnt5a was an important mediator for TAMs to support tumor, it did not directly affect CRC cells. Thus, Wnt5a mediated the pro-tumor effects of TAM through other soluble factors. We then analyzed the potential factors by RT-qPCR, which both facilitated cancer progression and increased the recruitment of macrophages 4, 20. We found that mRNA levels of CCL2 emerged as the most prominently upregulated and abundant cytokine in Wnt5a-treated M0 macrophages than M0 macrophages (Fig. 3A). Further ELISA analysis showed that CCL2 protein levels were significantly increased in the media from Wnt5a-treated M0 macrophages compared to M0 macrophages (Fig. 3B). Meanwhile, CCL2 expression in TAMs was also markedly higher than M0 macrophages, whereas Wnt5a knockdown reduced CCL2 expression in TAMs (Fig. 3A and B). To explore whether the pro-tumor functions of Wnt5a^+^ TAMs were mediated by CCL2, human recombinant CCL2 or neutralizing CCL2 antibody was used. CCL2 antibody treatment suppressed TAM-enhanced proliferation and migratory ability of HCT116 or DLD-1 cells (Fig. 3C-E). Consistent with previous results, the cancer-promoting capacity of TAMs was significantly impaired by Wnt5a knockdown, which was reversed by recombinant CCL2 treatment (Fig. 3C-E). In addition, the migration of THP-1 macrophages towards Wnt5a^+^ TAMs was also significantly inhibited by CCL2 neutralizing antibody, and the recruitment of THP-1 macrophages towards Wnt5a knockdown TAMs was enhanced by recombinant CCL2 (Fig. 3F and G). Collectively, these data suggest that Wnt5a mediates the cancer-promoting functions and macrophage recruitment of TAMs via CCL2.

### Wnt5a-CaMKII-ERK pathway is required for CCL2 expression in TAMs

**Figure 4:**
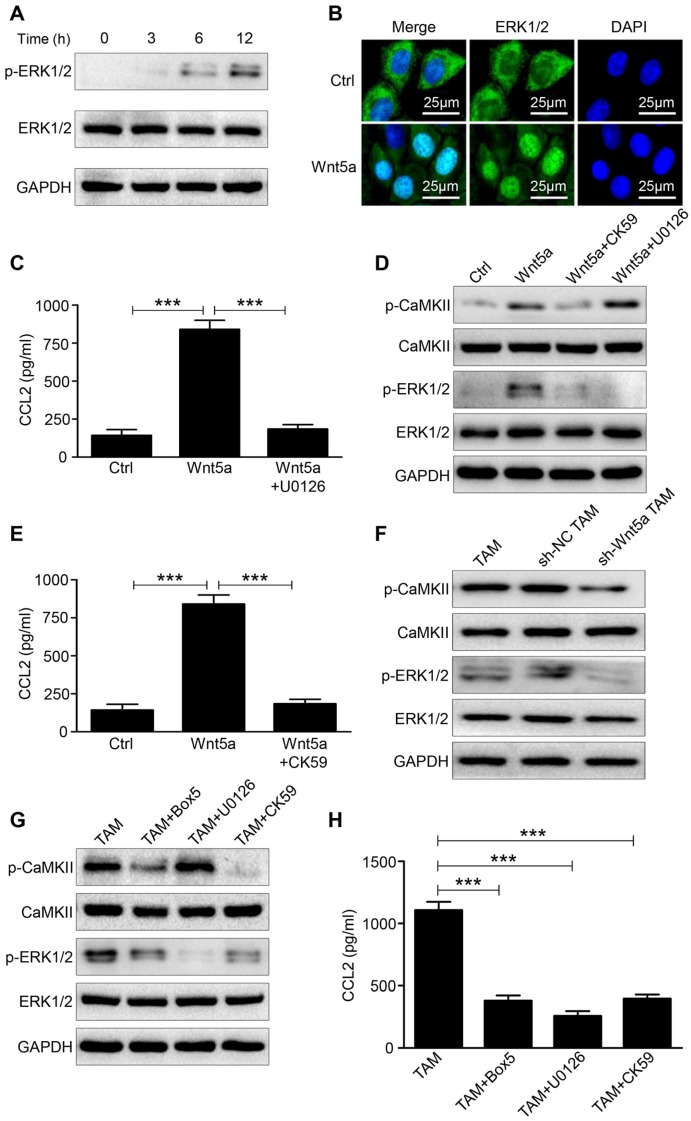
** Wnt5a regulates CCL2 expression through CaMKII-ERK1/2 pathway. A** Western blot analysis of p-ERK1/2 level at the indicated time in M0 macrophages treated with Wnt5a. **B** Immunofluorescence analysis for ERK1/2 in M0 macrophages treated with Wnt5a. Bar = 25μm. **C** ELISA analysis of CCL2 expression in Wnt5a-treated M0 macrophages with or without U0126. Error bars, SEM. **D** Western blot analysis of Wnt5a-treated M0 macrophages with or without CK59 or U0126. **E** ELISA analysis of CCL2 expression in Wnt5a-treated M0 macrophages with or without CK59. Error bars, SEM. **F** Western blot analysis of TAMs transfected with sh-Wnt5a or sh-NC. **G** Western blot analysis of TAMs with or without Box5 or U0126 or CK59. **H** ELISA analysis of CCL2 secretion level in TAMs with or without Box5 or U0126 or CK59. Error bars, SEM. ***P<0.001

Multiple studies suggested that the induction of CCL2 in various types of cells was regulated by ERK1/2 signaling pathway 21-23. Western blot was performed to determine the phosphorylation level of ERK1/2 after treating M0 macrophages with Wnt5a. The phosphorylation of ERK1/2 (p-ERK1/2) was increased within 6h after Wnt5a treatment (Fig. 4A). Further immunofluorescence analysis suggested that ERK1/2 was translocated to the nucleus of Wnt5a-treated M0 macrophages (Fig. 4B), thereby regulating CCL2 expression. Blocking p-ERK1/2 with the small-molecular inhibitor U0126 completely neutralized CCL2 induction by Wnt5a (Fig. 4C). Ca2^+^/CaMKII signaling pathway can be activated by Wnt5a, which then activates the downstream signaling pathway of MAPK-ERK1/2 24, 25. As shown in Fig. 4D, CaMKII was phosphorylated in Wnt5a-treated M0 macrophages. To investigate the regulatory relationship between CaMKII and ERK1/2, western blot was conducted to detect p-CaMKII and p-ERK1/2 expression in Wnt5a-stimulated M0 macrophages with or without CK59 (a small-molecular inhibitor of p-CaMKII) or U0126. The results indicated that p-ERK1/2 level induced by Wnt5a was entirely suppressed by CK59, while p-CaMKII expression level was hardly affected by U0126 (Fig. 4D). Meanwhile, CK59 also abolished Wnt5a-induced CCL2 expression (Fig. 4E).

We then knocked down the expression of Wnt5a in TAMs and found that the levels of p-CaMKII and p-ERK1/2 were both down-regulated (Fig. 4F). Furthermore, Wnt5a^+^ TAMs were treated with Box5 (Wnt5a specific inhibitor) or U0126 or CK59. As shown in Fig. 4G, Box5 could reduce p-CaMKII and p-ERK1/2 level. Consistent with the above results, CK59 could inhibit the phosphorylation level of ERK1/2, whereas U0126 hardly affected the phosphorylation level of CaMKII (Fig. 4G). In addition, CCL2 secretion level in Wnt5a^+^ TAM was suppressed by those inhibitors (Fig. 4H). Taken together, these data demonstrate that Wnt5a regulates CCL2 secretion through CaMKII-ERK1/2 pathway.

### Wnt5a^+^ TAM accelerates CRC tumorigenesis *in vivo*

**Figure 5:**
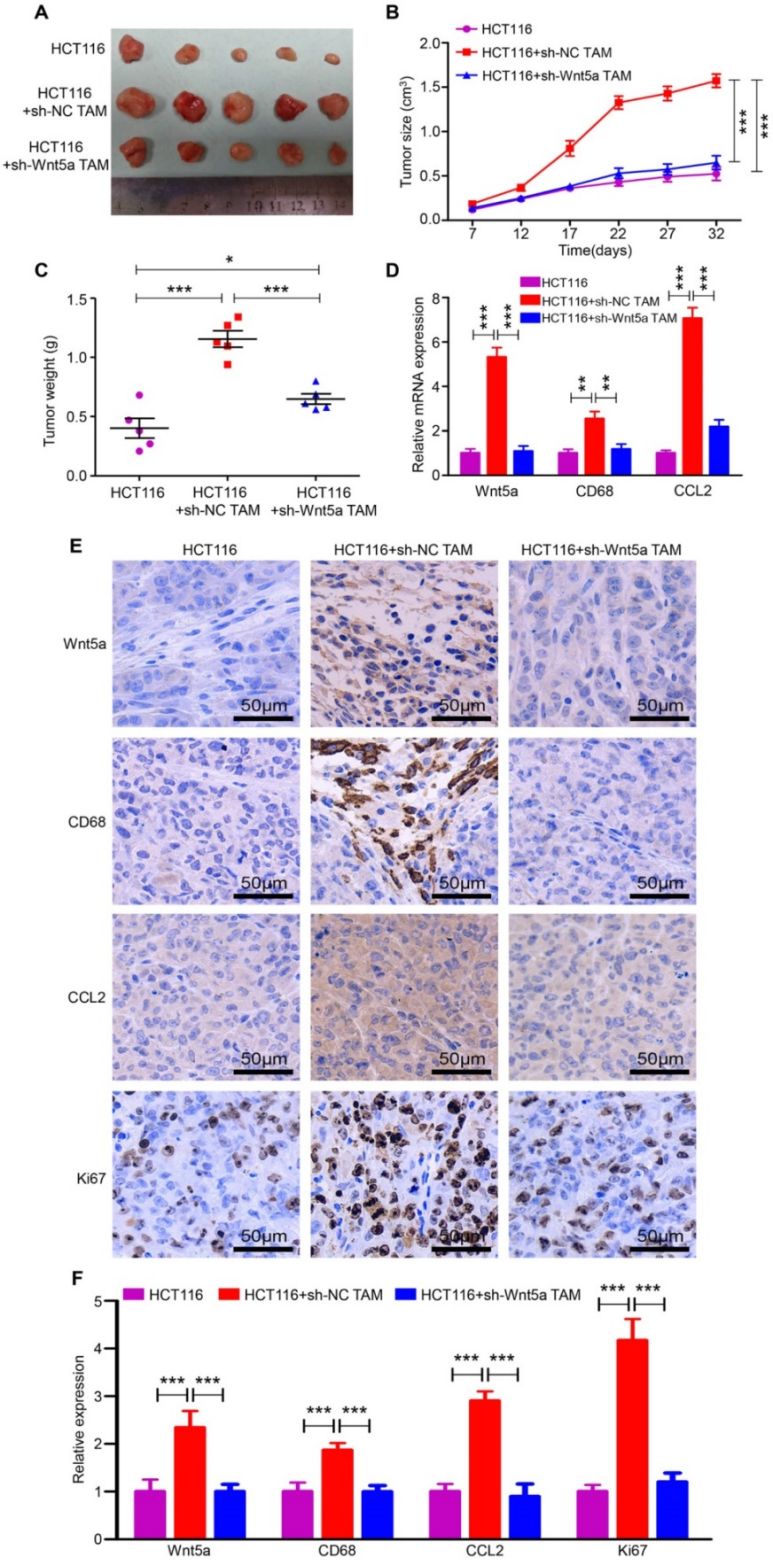
** Wnt5a knockdown impairs TAM-induced CRC tumorigenesis *in vivo*. A** The morphological characteristics of tumors in the HCT116 alone, HCT116+sh-NC TAM and HCT116+sh-Wnt5a TAM groups. **B** Volume of tumor growth at the indicated time. Error bars, SEM. **C** Tumor weight. Error bars, SEM. **D** Relative mRNA expression of Wnt5a, CD68 and CCL2 of tumors in different groups. Error bars, SEM. **E** IHC analyzed the expression of Wnt5a, CD68, CCL2 and Ki67 protein of tumors in different groups. Bar = 50μm. **F** Quantification analysis of Wnt5a, CD68, CCL2 and Ki67 protein expression measured by IHC. Error bars, SEM. **P< 0.01. ***P<0.001

To validate the above *in vitro* results, we applied an *in vivo* xenograft model. HCT116 cells, HCT116+sh-NC TAMs and HCT116+sh-Wnt5a TAMs were subcutaneously injected into the right flank of nude mice. After 32 days, tumors in the HCT116+sh-NC TAMs group were larger and heavier than that in the HCT116 alone group (Fig. 5A-C). However, tumors in the HCT116+sh-Wnt5a TAMs group were distinctly smaller and lighter than that in the HCT116+sh-NC TAMs group (Fig. 5A-C). Similarly, tumor cell proliferation was decelerated in mice injected with Wnt5a-silenced TAMs, as measured by Ki-67 staining (Fig. 5E and F). Moreover, RT-qPCR (Fig. 5D) and IHC staining (Fig. 5E and F) also confirmed that Wnt5a, CD68, and CCL2 expression levels were significantly elevated in the HCT116+sh-NC TAMs group, indicating more macrophages infiltration and CCL2 secretion. These data demonstrate that Wnt5a^+^ TAM enhances tumor growth and Wnt5a knockdown impairs TAM-induced CRC tumorigenesis *in vivo*.

## Discussion

In this study, we primarily found a novel TAM subtype, Wnt5a^+^ TAM, which played a vital role in tumor support. Further investigation demonstrated that Wnt5a was an important inducer of TAMs for their cancer-promoting roles. Mechanistically, the pro-tumor effects of Wnt5a in TAMs were dependent on CaMKII-ERK pathway-mediated CCL2 production.

Tumor-associated macrophages are one of the most dynamic immune cells in CRC, which are closely related to the occurrence and development of cancers 3, 26. Interestingly, TAMs are confirmed to have paradoxical roles in tumors. Some studies reported that TAM infiltration was associated with poor prognostic factor 27, 28, while others showed that TAM infiltration was correlated with better prognosis 29, 30. This discrepancy was particularly pronounced in colorectal cancer 27, 30. Previously, our studies had shown that CD163^+^ TAM subtype was a better predictor of clinical prognosis than pan-TAM 8, 31. Therefore, we thought that TAM subtype would be a better indicator for predicting tumor prognosis. Zhang et al. reported that high CD51^+^ TAM subtype infiltration was significantly correlated with poor clinical outcomes in pancreatic cancer 32. Cortes and colleagues showed that ZEB1^+^ TAMs were associated with tumor growth, poor prognosis, and chemotherapy resistance in ovarian carcinoma 33. In this work, we also demonstrated that Wnt5a^+^ TAM was a tumor-supporting TAM subtype. These findings reveal that the Wnt5a^+^ TAM subtype could be a preferable marker of CRC prognosis to pan-TAM.

We found that Wnt5a was overexpressed in TAMs rather than in CRC cells. *In vivo* and *in vitro* experiments demonstrated that Wnt5a^+^ TAMs facilitated CRC growth. Wnt5a knockdown mostly reduced the cancer-promoting functions of TAMs, showing that TAMs were dependent on Wnt5a for their pro-tumor roles. A few studies reported that Wnt5a had scarce or low expression in CRC and played an anti-tumor role in cancer development 34, 35. Their studies mainly focused on the direct roles of Wnt5a on CRC cells. But in fact, Smith et al. found that Wnt5a was mostly expressed on TAMs in tumor microenvironment of CRC 16, which is similar to our findings. Nitzki and colleagues also reported that Wnt5a was primarily derived from TAMs of the tumor stroma in basal cell carcinoma 36. Furthermore, our *in vitro* experiments had also revealed that direct stimulation with Wnt5a insignificantly influenced the malignant behaviors of CRC cells. Therefore, we believed that Wnt5a was more prone to indirectly facilitate tumor progression through TAMs.

In fact, the CD51^+^ TAM or ZEB1^+^ TAM subtype mentioned above was an M2-like phenotype 32, 33. Here we identified that Wnt5a^+^ TAM was also a phenotype of M2-like TAM. Bergenfelz et al. also found that Wnt5a was mainly co-localized with CD163 (M2 marker) 37. However, the regulatory relationship between Wnt5a and M2-like TAMs needs further exploration. Further studies are also necessary to focus on the mechanism by which Wnt5a expression is increased on TAMs in CRC. Recently, some studies have shown that Wnt/β-catenin signaling pathway plays an important role in regulating M2 polarization of TAMs. Feng et al. found that Wnt/β-catenin signaling was crucial for IL-4/TGFβ1-induced M2 polarization of macrophage 38. Yang and colleagues reported that Wnt/β-catenin signaling was involved in M2-like TAM polarization by regulating c-Myc, and knockdown of β-catenin in M2 TAMs suppressed the tumor-promoting functions of TAMs^39^. Although canonical Wnt/β-catenin signaling has been found to regulate M2-like TAM polarization, aberrant Wnt/β-catenin signaling in CRC, including aberrant APC and β-catenin, mainly occurs in cancer cells and affect the biological behaviors of tumor cells 40, 41. While aberrant noncanonical Wnt signaling pathway may be more likely to present in the tumor microenvironment of CRC. For example, Wnt5a, mainly activating the non-canonical Wnt pathways such as planar cell polarity (PCP), JNK and Ca2^+^/CaKMII pathways^11^, ^12^, was found to be located in M2-like TAM of tumor stroma and regulated the biological function of M2-like TAM in this study. Wnt5a could also remodel tumor microenvironment by inducing a fibroblast-like or mesenchymal phenotype of adipocytes, which then favors tumor development 42. Therefore, canonical Wnt/beta-catenin pathway and noncanonical Wnt pathways are both involved in the regulation of CRC progression.

Multiple research mainly tended to M2-like TAM, which was closely related to poor prognosis in CRC 3, 7. M2-like TAMs drive tumor progression and development through secreted proteins such as CCL2, TNF-α, IL-6, TGF-β and EGF, which promote tumor invasiveness 2, 6, 20. Wnt5a^+^ TAM also recruited more monocyte-macrophage infiltration beyond its supporting role in CRC. Therefore, we evaluated potential important secretory factors and found that the expression of CCL2 was the most significantly upregulated after Wnt5a treatment. It had been reported by various studies that CCL2 not only promoted tumor invasion and metastasis, but also acted as a chemokine to attract TAMs infiltration 20, 43. Further experiments revealed that the cancer-promoting functions of Wnt5a in TAMs were dependent on CCL2 secretion.

Mechanistically, we demonstrated that CaMKII-ERK pathway was essential for CCL2 production induced by Wnt5a in TAMs. Here we confirmed that Wnt5a could activate ERK signaling pathway to regulate CCL2 expression. Several studies had also suggested that CCL2 was regulated by ERK pathway 21-23. Wnt5a, a member of Wnt family, is a secreted protein, which primarily activates the non-canonical pathways such as planar cell polarity (PCP) and Ca2^+^/CaKMII pathway through binding to frizzled receptor and LRP co-receptors 11, 15. So, we further found that Ca2^+^/CaKMII pathway was also activated by Wnt5a in macrophages. As one of the downstream pathways of Ca2^+^/CaKMII 24, 25, the MAPK-ERK pathway was suppressed when Ca2^+^/CaKMII pathway was deactivated by inhibitor in Wnt5a-stimulated macrophages, thereby suppressing CCL2 production.

In conclusion, we demonstrated that Wnt5a was mainly localized in TAMs of tumor stroma, especially M2-like TAMs. Wnt5a^+^ TAMs played an important role in malignant biological behavior and facilitating macrophages infiltration partially due to paracrine secretion of CCL2. The tumor-promoting functions of Wnt5a in TAMs depended on CaMKII-ERK pathway-mediated CCL2 secretion. Our data not only report a novel Wnt5a^+^ TAM subtype and its pro-tumor roles in CRC, but also provide a novel insight into targeting Wnt5a in cancer therapy.

## Supplementary Material

Supplementary figure and table.

## Abbreviations

TME: tumor microenvironment
TAMs: tumor-associated macrophages
CRC: colorectal cancer
ERK: extracellular regulated protein kinases
CaMKII: calmodulin-dependent protein kinase II
IHC: immunohistochemistry
