# Supplementary material for: Wnt5a/CaMKII/ERK/CCL2 axis is required for tumor-associated macrophages to promote colorectal cancer progression
Source: Int J Biol Sci. 2020 Feb 4;16(6):1023–34. doi: 10.7150/ijbs.40535 (PMC7053330; doi:10.7150/ijbs.40535)
Supplement: Supplementary file 1 — Supplementary figure and table. [file ijbsv16p1023s1.pdf]

Table S1 The sequences of the primers for quantitative RT-PCR

Genes	Primer Sequence (5' to 3')	Product size (bp)
HLA-DR	F: TCTGGCGGCTTGAAGAATTTG<br>R: GGTGATCGGAGTATAGTTGGAGC	125
CD86	F: GCCTTCCTGCTCTCTGCTAA<br>R: TCTTAGGTTCTGGGTAACCGTG	118
IL-12	F: ACCTGACCCACCCAAGAACT<br>R: GGACCTGAACGCAGAATGTC	131
IL-23	F: CCCAAGGACTCAGGGACAAC<br>R: AGAGAAGGCTCCCCTGTGAA	109
CD163	F: TTGTCAACTTGAGTCCCTTCAC<br>R: TCCCGCTACACTTGTTTTCAC	127
CD206	F: GGGTTGCTATCACTCTCTATGC<br>R: TTTCTTGTCTGTTGCCGTAGTT	126
Arg-1	F: TGGACAGACTAGGAATTGGCA<br>R: CCAGTCCGTCAACATCAAAACT	102
IL-10	F: GCCAAGCCTTGTCTGAGATGATCC<br>R: TTCACATGCGCCTTGATGTCTGG	91
TGF-β	F: AAGGACCTCGGCTGGAAGTGC<br>R: CCGGGTTATGCTGGTTGTA	136
CCL17	F: CTTCAAGGGAGCCATTCCCC<br>R: CTCTTGTTGTTGGGGTCCGA	129
CCL18	F: CTCTGCTGCCTCGTCTATACCT<br>R: CTTGGTTAGGAGGATGACACCT	108
CCL22	F: CCGCTCTGCAGGGTATTTGA<br>R: GCCCCACAGCAAGCCTATAA	111
Wnt5a	F: GTTTCGGCTACAGACCCAGA<br>R: CCCCAGTTCATTCACACCACA	92
CSF-1	F: AGCCAGAAGGAGGACCAGCAAG<br>R: ACCAGCAGGTGGAAGACAGACTC	145
VEGF	F: GAGGAGCAGTTACGGTCTGTG<br>R: TCCTTTCCTTAGCTGACACTTGT	96
CCL2	F: CATCTCCTACACCCCACGAAG<br>R: GGGTTGGCACAGAAACGTC	126
CCL5	F: CCAGCAGTCGTCTTTGTCAC<br>R: CTCTGGGTTGGCACACACTT	54
CD68	F: GGGCTACTGGCAGAGAGCA<br>R: GTTGAGGGTCCCTGGCTG	200
GAPDH	F: GCACCACCAACTGCTTAGCA<br>R: GTCTTCTGGGTGGCAGTGATG	106

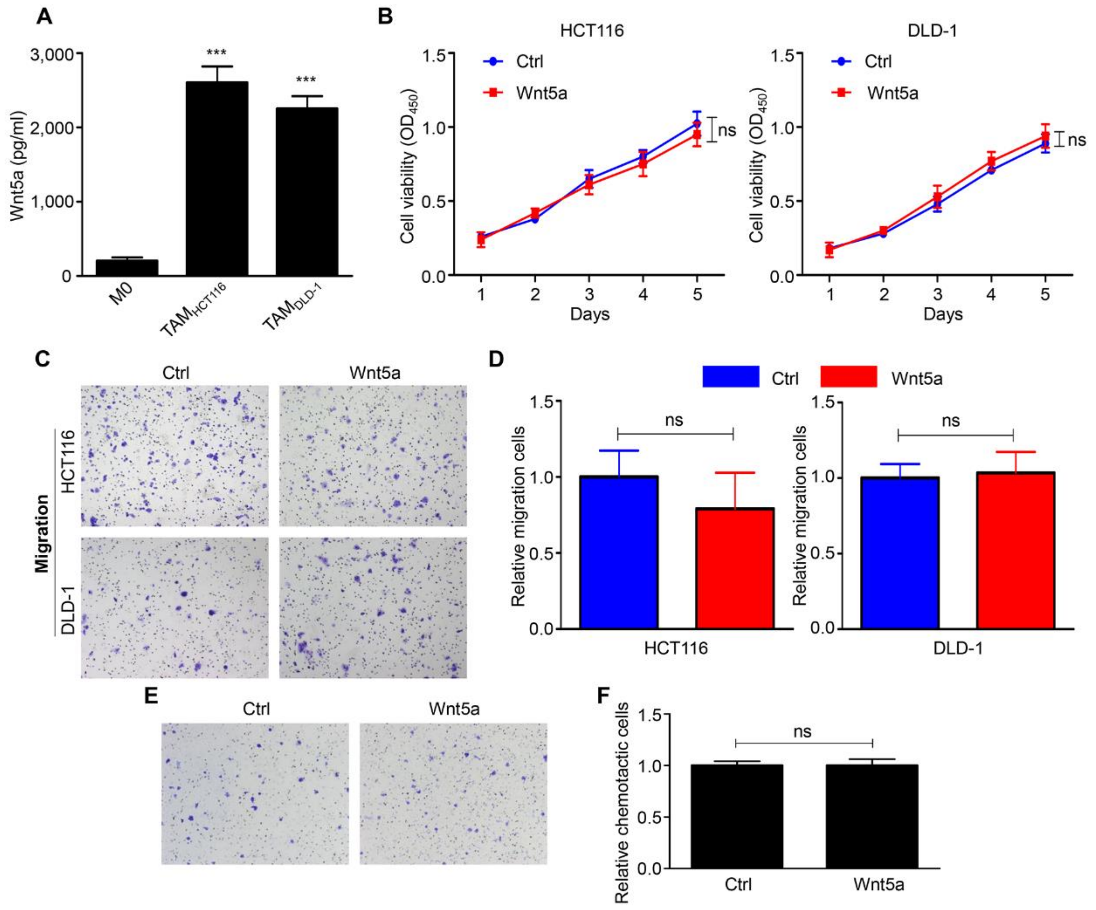

Figure S1. Wnt5a does not directly influence CRC cells and macrophage recruitment. **A** ELISA analysis of Wnt5a secretion level in M0 macrophages and TAMs. Error bars, SEM. **B** Cell viability of HCT116 or DLD-1 cells treated with Wnt5a. Error bars, SEM. **C** Transwell migration assay of HCT116 and DLD-1 cells treated with Wnt5a. (magnification, ×100). **D** Quantification analysis of migratory cells in five fields was counted manually. Error bars, SEM. **E** Chemotaxis analysis of THP-1 macrophages toward Wnt5a. (magnification, ×100). **F** Quantification analysis of chemotactic cells in five fields was counted manually. Error bars, SEM. ns, not significant. ***P<0.001
